# Integrated Analysis of lncRNA and mRNA in Subcutaneous Adipose Tissue of Ningxiang Pig

**DOI:** 10.3390/biology10080726

**Published:** 2021-07-29

**Authors:** Yan Gong, Jun He, Biao Li, Yu Xiao, Qinghua Zeng, Kang Xu, Yehui Duan, Jianhua He, Haiming Ma

**Affiliations:** 1 College of Animal Science and Technology, Hunan Agricultural University, Changsha 410128, China; 13910008175@139.com (Y.G.); hejun@hunau.edu.cn (J.H.); 18874028597@163.com (B.L.); xiaoyu1030189228@126.com (Y.X.); zhouwei_2005@126.com (Q.Z.); jianhuahy@hunau.net (J.H.); 2 Ningxiang Pig Farm of Dalong Livestock Technology Co., Ltd., Ningxiang 410600, China; 3 Laboratory of Animal Nutritional Physiology and Metabolic Process, Key Laboratory of Agroecological Processes in Subtropical Region, Institute of Subtropical Agriculture, Chinese Academy of Sciences, Changsha 410125, China; xukang2020@isa.ac.cn (K.X.); duanyehui@isa.ac.cn (Y.D.)

**Keywords:** Ningxiang pig, subcutaneous adipose tissue, mRNAs, lncRNAs, STEM, WGCNA

## Abstract

**Simple Summary:**

This study shows the transcription profiles and the functional network in lncRNA and mRNA in the subcutaneous adipose tissue of Ningxiang piglets in four stages of development (piglets, nursery pigs, early fattening, and late fattening). A total of 2872 novel lncRNAs have now been determined. A total of 10,084 DEmRNAs and 931 DElncRNAs were determined. Interestingly, most DEmRNAs were up-regulated in the piglet stage and, in contrast, DElncRNAs were up-regulated in the late fattening stage. A complicated interaction between mRNAs and lncRNAs was determined via STEM and WGCNA, demonstrating that lncRNAs are an essential regulatory component in mRNAs. Modules 2 and 5 shows a similar mode of transcriptions for both mRNA and lncRNA, which are mainly involved in steroid biosynthesis, glycosphingolipid biosynthesis, metabolic pathways, and glycerolipid metabolism. The transcription levels of mRNAs and lncRNAs for both modules were higher in the early and late fattening stage. This may be explained by the active fatty acids, sterols, steroids, and lipid-related metabolic activity in the subcutaneous adipose tissue during the early and late fattening stage.

**Abstract:**

Ningxiang pigs, a Chinese bred pig known for its tender meat and high quality unsaturated fatty acids. This study discovers the transcription profiles and functional networks in long non-coding RNA (lncRNA) and messenger RNA (mRNA) in subcutaneous adipose tissue. Subcutaneous adipose tissue was collected from piglet, nursery pig, early fattening, and late fattening stage of Ningxiang piglets, and lncRNA and mRNA transcription of each stage was profiled. A total of 339,204,926 (piglet), 315,609,246 (nursery), 266,798,202 (early fattening), and 343,740,308 (late fattening) clean reads were generated, and 2872 novel lncRNAs were identified. Additionally, 10,084 differential mRNAs (DEmRNAs) and 931 differential lncRNAs were determined. Most DEmRNAs were up-regulated in the piglet stage, while they were down-regulated in late fattening stage. A complicated interaction between mRNAs and lncRNAs was identified via STEM and WGCNA, demonstrated that lncRNAs are a significant regulatory component in mRNAs. The findings showed that modules 2 and 5 have a similar mode of transcription for both mRNA and lncRNA, and were mainly participated in steroid biosynthesis, glycosphingolipid biosynthesis, metabolic pathways, and glycerolipid metabolism. The mRNAs and lncRNAs transcription levels of both modules was higher in the early and late fattening stage, which may be due to the active activity of the metabolism in relation to fatty acids, sterols, steroids, and lipids in the subcutaneous adipose tissue during the early and late fattening stage. These findings could be expected to result in further research of the functional properties of lncRNA from subcutaneous adipose tissue at different stages of development in Ningxiang pigs.

## 1. Introduction

There are more than 100 breeds of pigs in China, and one-third of the world’s pig breeds are found in China [1]. Ningxiang pig, also called Caochong pig or Liusha-He pig [2], is a famous Chinese pig breed in China and is native to Hunan Province. Ningxiang pigs have a breeding history of more than a thousand years in China. They are renowned for their tender and juicy meat, their unique taste, and the high quality of unsaturated fatty acids. Because of their breeding history and advantages, Ningxiang pigs have become a significant livestock resource and a geographical representation of the Chinese agricultural product [3]. At the same time, Ningxiang pigs have become a miniature experimental pig line by scientific research institutions, since miniature pigs are very close to humans in terms of anatomy and physiology [4]. Compared to other pig species, Ningxiang pigs have more than 5% of intramuscular adipose tissues [5,6]. High intramuscular fat content improves the meat quality and taste in Ningxiang pigs. In addition, Ningxiang pigs showed better disease resistance [7] and pronounced genetic characteristic such as strong adaptability and high reproductive capacity [2].

As we know, the genetic information of the organism was deposited in the protein-coding genes [8]; therefore, for a long period of time, RNA was only regarded as an intermediary between the DNA sequence and its encoded protein [9]. In addition, there is increasing evidence that non-coding RNA (ncRNA) is an important element in many biological processes. In particular, long non-coding RNA (lncRNA) is an important type of ncRNA with a length of more than 200 nucleotides (nt) [10]. Many lncRNAs have been recognized as a rapid development of experimental technologies and computational methods [11]. However, a previous study reported that transcription lncRNAs levels were lower than the protein-coding genes [12]. Ample evidence showed that lncRNAs were participated in a broad range of biological processes, such as RNA processing, epigenetic regulation, chromatin regulation, gene transcription, translation, splicing, and cell cycle control [13]. The lncRNA is a complex and diverse ncRNA that is related to introns and exons as well as some features of mRNA [14]. LncRNAs have shown the ability to regularize gene expression through miscellaneous mechanisms, including complementary binding to protein coding transcripts in the cis-trans lncRNA manner [15]. In mammals, however, the regulatory relationship to the corresponding potential target genes, particularly in the biosynthesis, transport, and metabolism of fatty acids, remains unclear. It is therefore meaningful to discover the regulation of the molecular mechanisms of lipid metabolism in subcutaneous adipose tissue. LncRNA, especially in the transcription process, has grown to be a hot topic in recent years. Therefore, in this study, we investigate the regulation of lncRNA in the mRNA of Ningxiang pigs in four stages of development.

Pork is one of the primary sources of protein and lipids for human and accounts for about 30% of global meat consumption [16]. The subcutaneous adipose tissue in the back of Ningxiang pigs is rich in oleic acid, palmitic acid, stearic acid, and linoleum acid, which are essential fatty acids for humans [3]. Fat deposition in subcutaneous adipose tissue and fat traits is one of the essential characteristics in pigs as they are closely associated with human nutritional value [17]. The quantity of fat deposited in subcutaneous fat is critical to growth performance. Pigs have a better growth performance when less fat is deposited. As an advance in the next generation sequencing, many researchers used the RNA-seq technology to study the transcriptomic profile of various adipose tissues in different breeds of pigs [18,19,20]. In this study, we systematically discovered and identified the lncRNAs and mRNAs of the subcutaneous adipose tissue at all four different developmental stages, including the piglet stage (30 days after birth), nursery pig stage (90 days after birth), early fattening stage (150 days after birth), and late fattening stage (210 days after birth) through high-throughput sequencing technology. The results of this study provide fundamental insights and references for studying the function and mechanisms of lncRNAs in subcutaneous adipose tissue. It can also serve as a meaningful reference for studies on other Chinese pig breeds. In addition, it can provide molecular information for further research on Ningxiang pigs. These findings may offer a crucial data for research on nutrition, feed management, and actual production of Ningxiang pigs. 

## 2. Materials and Methods

### 2.1. Ethics Statement

The treatment and handling protocol for Ningxiang pigs was conducted according to the Laboratory Animals—Guideline of Welfare and Ethics of China. The experimental protocols in this study were approved by the Institutional Animal Care and Use Committee of Hunan Agricultural University, Changsha, Hunan Province, China, with the approval number of No. 2013-06. 

### 2.2. Experimental Animal and Sample Collection

Half-sibling piglets were obtained by mating one male Ningxiang pig with four female Ningxiang pigs. Twelve tails of half-sibling Ningxiang piglet were obtained from the Hunan Chuweixiang Agriculture and Animal Husbanary Co. Ltd. (Ningxiang, Hunan, China). In this experiment, they were raised, fed, and treated in the same way and in the same environment from birth. Twelve healthy full-sibling piglets were randomly chosen for slaughter to collect subcutaneous adipose tissue samples in four developmental stages: piglet stage (30 days after birth), nursery pig stage (90 days after birth), early fattening stage (150 days after birth), and late fattening stage (210 days after birth); and three tails piglets were randomly selected to be slaughtered for each stage of development. Subcutaneous adipose tissue samples were obtained from the piglet at the same location in each animal at each collection time point. Subcutaneous adipose tissue samples were collected and immediately stored in liquid nitrogen (−196 °C), and then kept in a freezer at (−80 °C) for RNA isolation. The samples were stored in ultra-low temperature to avoid RNA degradation. 

### 2.3. RNA Extraction

The total RNA from the subcutaneous adipose tissue was extracted using TRIzol reagent (TaKaRa Bio. Inc., Dalian, China) according to the manufacturer’s protocols. The extracted total RNAs were then processed by RNase-free DNase to remove the excess DNA. Then, the quality of extracted RNAs from subcutaneous adipose tissue was evaluated by Nanodrop 2000 (Thermo Fisher Scientific, Waltham, MA, USA) and assessed by 1% agarose gel electrophoresis. Qualified total RNAs were stored at −80 °C until use. 

### 2.4. Library Construction and RNA-Seq 

An amount of 5 μg of total RNA from subcutaneous adipose tissue samples was used to prepare RNA-seq transcriptome strand library using TruseqTM stranded total RNA kit (Illumina, San Diego, CA, USA). Ribosomal RNA depletion instead of poly A purification was performed with the Ribo-Zero Magnetic kit and then fragmented with fragmentation buffer. First-strand cDNA was generated with random hexamer primers. RNA templates were then eliminated to make a replacement strand, containing dUTP for the synthesis of double-stranded cDNA (dscDNA). AMPure XP beads were used to extract the dscDNA from the second strand reaction mix. A single ‘A’ nucleotide was added to the 3’ ends of the blunt fragments to prevent them from ligating together during the adapter ligation process and, eventually, multiple indexing adapters were ligated at the ends of the dscDNA. The sizes of libraries were selected for cDNA with a target fragment size of 200–300bp in 2% low range ultra-agarose, followed by PCR amplification using Phusion DNA polymerase (NEB, Ipswich, MA, USA) for 15 cycles. A pair-end RNA-seq sequencing library was sequenced with the Illumina Hiseqxten (2 × 150 bp read lengths) after TBS380 quantification.

### 2.5. Read Mapping and Transcriptome Assembly

Raw sequencing data contains low quality reads, sequences with high N rate, and sequences that are very short in length, all of which will severely influence the quality of subsequent analysis. To ensure the quality and accuracy of subsequent biological information analysis, the raw sequencing data were first filtered to obtain high quality clean data in order to ensure the quality and accuracy of subsequent biological information analysis. SeqPrep (https://github.com/jstjohn/SeqPrep, accessed on 2 November 2020) and Sickle (https://github.com/najoshi/sickle, accessed on 5 November 2020) were used to access and trim the quality of the sequencing reads. During trimming, reads with linker sequence and read without inserted fragments due to self-connection were eliminate; sequences with quality value less than 20 (low-quality) at the 3’ end was trimmed off; reads with quality values less than 10 in the remaining sequences was discarded; and reads with more than 10% of N were discarded. We mapped sequences using TopHat2 [21]. The obtained cleans reads were mapped and aligned against the Ningxiang pig reference genome (the accession number: PPJNA531381, the Ningxiang pigs draft genome is still confidential and has not yet been unpublished). The reads were split into smaller fragments and aligned to the reference genome.

### 2.6. Identification and Classification of LncRNAs

In the preliminary screening of lncRNAs, the cuffcompare program in the Cufflinks suite was used to screen the intergenic, intronic, and anti-sense lncRNAs. Transcripts with fragment count ≤ 3, transcripts shorter than 200 nt, open reading frame (ORF) longer than 300 nt, and an exon number < 2 were used as screening criteria for lncRNAs. For those transcripts with the fragment count ≤ 3, transcripts shorter than 200 nt, ORF longer than 300 nt, and exon number < 2 were removed. Being the initial screening, preliminary screening was performed to screen for the coding potential of the lncRNAs. Transcripts with coding potential were filtered by Coding Potential Assessment Tool (CPAT), Coding-Non-Coding Index (CNCI), and Coding Potential Calculator (CPC). The remaining transcripts with identified protein domains were excluded by Pfam Scan under Pfam HMM. The remaining transcripts were considered reliably expressed as lncRNAs. The expression level of each lncRNA was determined using the fragments per kilobase of exon per million mapped reads (FRKM) method. The significantly differently expressed (DE) lncRNAs were extracted with |log2FC| > 1, false discovery rate (FDR) < 0.05 by edgeR.

### 2.7. Functional Enrichment and Differential Expression Analysis

Adapter sequences were removed from the raw sequencing reads prior to alignment (as not removing, the read mapping percentage will be affected). Differential expression genes (DEGs) among the test samples were designated by the expression level of each transcript. Fragments per kilobase of exon per million mapped reads (FRKM) was used to calculate the expression level of each transcript. RSEM (http://deweylab.biostat.wisc.edu/rsem/, accessed on 10 November 2020) was used to quantify gene abundance. Corresponding group: 30-day vs. 90-day, 30-day vs. 150-day, 30-day vs. 210-day, 90-day vs. 150-day, 90-day vs. 210-day, and 150-day vs. 210-day were assigned to compare differential expression. Differential expression for each gene was analyzed by the Empirical Analysis of Digital Gene Expression in R (edgeR) software in the R statistical package. Furthermore, functional enrichment analysis including Gene Ontology (GO) and the Kyoto Encyclopedia of Genes and Genomes (KEGG) was performed to determine which DEGs were significantly enriched in GO terms and metabolic pathways compared to whole transcriptome background at a Bonferroni-corrected *p*-value ≤ 0.05. Goatools and KOBAS were used to analyze GO functional enrichment and KEGG pathway. 

### 2.8. Time Series Analysis (Short Time-Series Expression Miner, STEM)

To investigate the relationship between temporal gene expression patterns and subcutaneous adipose tissue at 4 piglet development stages, a Short Time-Series Expression Miner (STEM) clustering algorithm was used to classify protein-coding genes and lncRNA expression profiles [22]. The novel clustering method implemented by STEM was first defined as a set of distinct and representative model temporal expression profiles independent of the data. These model profiles correspond to possible profiles of gene expression versus time. The model profiles start at 0, and then will remain stable between two time points, or increase or decrease in an integral number of time units up to a parameter value. The number of genes determined for each model profile is the computed value. The number of genes expected to be assigned to a profile is estimated by randomly permuting the original time point values, renormalizing the gene expression values, then assigning genes to the closest-matching model profiles, and repeating the order for multiple times. The true order of time points was used to test the standard hypothesis and *p*-value were estimated from the number of genes in the model profile and the number of assigned genes (adjusted *p*-value ≤ 0.05 by Bonferroni correction). Colored model profiles (other than white) indicate statistically significant temporal trends in mRNAs and lncRNAs. Profiles with the same color were grouped into the same cluster.

### 2.9. Co-Expression Network (Weight Correlation Network Analysis, WGCNA)

The mRNA–lncRNA co-expression networks were constructed through the developmental phases of the weighted correlation network analysis (WGCNA) package of R software [23]. After removing samples with outliers, the Pearson’s correlation coefficient between any two genes in the gene set was established and a correlation coefficients matrix was constructed. The appropriate threshold (β value) was selected to measure the weighted power exponent of the correlation coefficient matrix to construct the adjacency matrix. A topological overlap matrix was then developed and employed to the connection between genes. Gene modules were initially divided by hierarchical clustering analysis to obtain eigengenes according to related traits. Based on the similarity of eigengenes, the modules were merged to form the final module for further analysis [24].

### 2.10. Real-Time PCR Quantification

In order to confirm the reliability of RNA-seq data in Ningxiang pig, few lncRNA and mRNA genes were randomly selected for rt-qPCR quantification. The total RNA of subcutaneous adipose tissue was extracted using an animal Total RNA Kit (Tiangen, Beijing, China) and treated with ribonuclease R to validate the identified lncRNAs in Ningxiang pig. cDNA was synthesized by reverse transcription using the Revert Aid First Strand cDNA Synthesis Kit (Thermo Scientific, Waltham, MA, USA). The transcription levels of lncRNAs (MSTRG.1053.3, MSTRG.1054.2, MSTRG.11451.1, MSTRG.12045.1, and MSTRG.25924.3) and mRNAs (*GRHPR*, *FABP1*, *ACADSB*, *ELOVL5*, *FADS2*) were verified by Quanstudio 6 Flex (Applied Biosystems, Foster City, CA, USA). The forward and reverse primers for gene quantification are presented in Table 1. RT-qPCR was performed in a 96-well plate, with each well containing 20 μL of mixture, including 10 μL of SYBR Premix Ex Taqm II (TaKaRa, Dalian, China), 0.4 μL (10 μM) of forward and reverse primer, 2 μL of cDNA template, and 7.2 μL of diethyl pyrocarbonate (DEPC) water. The RT-qPCR running conditions were set as follows: 95 °C for 5 minutes (pre-denaturation) and 40 cycles of amplification (95 °C for 15 s, 59 °C for 40 s, and 72 °C for 20 s). The gene validation for each time point was made in triplicate. The expression level of each validated gene for each time point was calculated by 2^−^^ΔΔCt^ method.

## 3. Results

### 3.1. Identification and Classification of lncRNA in Ningxiang Pig Subcutaneous Adipose Tissue

**Figure 1:**
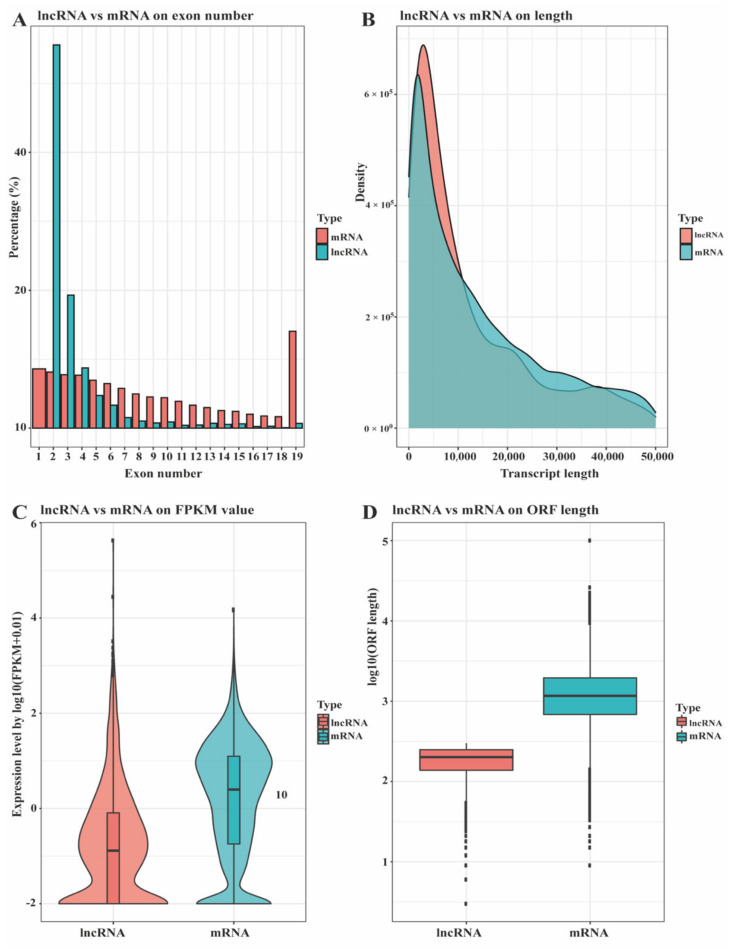
Genomic classification of lncRNA and mRNA transcripts was compared by (**A**) exon number, (**B**) length, (**C**) fragments per kilobase of exon per million mapped reads (FPKM) value, and (**D**) open reading frame (ORF) length.

The lncRNA and mRNA analysis subcutaneous adipose tissue from Ningxiang pigs at 30, 90, 150, and 210 days postnatal yield 339,204,926; 315,609,246; 266,798,202; and 343,740,308 clean reads, respectively, with Q30 of over 94.36% (Table S1). These clean reads were aligned to the Ningxiang pig reference genome (accession number: PPJNA531381) at rates ranging from 91.96 to 94.74% (Table S2). A total of 2872 novel lncRNAs were identified, and these were classified into five types: intergenic (1263), antisense (766), bidirectional (29), sense exon overlapping (769), and sense intron overlapping lncRNAs (45) (Table S3). The molecular characteristics of the identified novel lncRNA in subcutaneous adipose tissue were the same as previous results in mammals [25]. The lncRNA had a predominant concentration of two exons, followed by three exons (Figure 1A). Compared to mRNA transcripts, lncRNAs were shorter in length and their expression was less than that of mRNA (Figure 1B,C). Furthermore, the open reading frame length of lncRNA was also shorter than that of mRNA (Figure 1D).

### 3.2. Analysis of Differentially Expressed mRNA and lncRNAs

A total of 10084 differential mRNAs (DEmRNAs) (Table S4) and 931 differential lncRNAs (DElncRNAs) (Table S5) were identified through pairwise comparison of six groups (*p* < 0.05) in the subcutaneous adipose tissue of four developmental stages including piglet stage (30 days after birth), nursery pig stage (90 days after birth), early fattening stage (150 days after birth), and late fattening stage (210 days after birth). Among the DEmRNAs, the 150d vs. 210d had the lowest number of DEmRNAs (1334), followed by 90d vs. 150d group (3264); the 30d vs. 150d group had the highest number of DEmRNAs (7091), followed by the 30d vs. 210d group (6320). For the DElncRNAs section, 90d vs. 210d groups had the lowest number of DElncRNAs (171), while the 30d vs. 150d group had the highest number of DElncRNAs (751). 

### 3.3. Expression Patterns and GO Enrichment Analysis of mRNAs and lncRNAs

To discover the expression patterns of mRNAs in the subcutaneous adipose tissue of piglets at different development stages, two comparative methods were constructed, namely, closed group (30d vs. 90d, 30d vs. 150d, and 30d vs. 210d), and the consecutive groups (30d vs. 90d, 90d vs. 150d, and 150d vs. 210d). In the closed group, the number of up-regulated genes was 2792, 3429, and 3045 for 30d vs. 90d, 30d vs. 150d, and 30d vs. 210d, respectively; while the number of down-regulated genes was 2963, 3662, and 3275 for 30d vs. 90d, 30d vs. 150d, and 30d vs. 210d respectively. GO enrichment analysis for the closed groups is shown in Table 2. In the consecutive groups, the number of up-regulated genes was 2792, 1397, and 732 for 30d vs. 90d, 90d vs. 150d, and 150d vs. 210d, respectively; while the down-regulated genes were 2963, 1867, and 602 for 30d vs. 90d, 90d vs. 150d, and 150d vs. 210d respectively. GO enrichment analysis for the consecutive groups is shown in Table 3.

To discover the transcription patterns of lncRNAs in subcutaneous adipose tissue at different development stages in piglets, two comparative methods were constructed, namely, closed group (30d vs. 90d, 30d vs. 150d, and 30d vs. 210d), and consecutive groups (30d vs. 90d, 90d vs. 150d, and 150d vs. 210d). In the closed group, the number of up-regulated genes was 93, 111, and 97 for 30d vs. 90d, 30d vs. 150d, and 30d vs. 210d, respectively; while the number of down-regulated genes was 205, 640, and 190 for 30d vs. 90d, 30d vs. 150d, and 30d vs. 210d, respectively. GO enrichment analysis for the closed groups was shown in Table 2. In the consecutive groups, 30d vs. 90d, 90d vs. 150d, and 150d vs. 210d had 93, 54, and 382 up-regulated genes, respectively; while 30d vs. 90d, 90d vs. 150d, and 150d vs. 210d had 205, 351, and 23 down-regulated genes, respectively. GO enrichment analysis for the consecutive groups is shown in Table 3.

**Figure 2:**
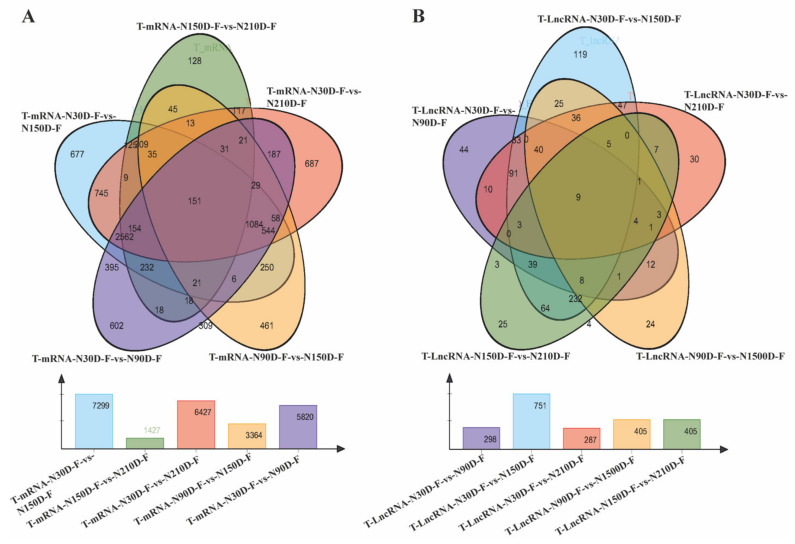
Venn diagram of (**A**) DEmRNAs and (**B**) DElncRNAs of subcutaneous adipose during 4 developmental stages with 3 closed and 2 consecutive groups.

The intersection of the closed and continuous group was used to identify differential mRNA and lncRNA common to the subcutaneous adipose tissue of Ningxiang pigs at four developmental stages, presented by a Venn diagram. As showed in Figure 2A,B, a total of 151 differential mRNAs and 9 differential lncRNAs were identified. Among the common differential mRNAs, we found that *FADS2*, *CYP3A29*, *CYP2C42*, *AGMO*, *PLCB4*, *LEPR*, *CES1D* participated in lipid metabolism, while *IGF2*, *IGFALS*, and *FGFR4* were involved in cell differentiation and proliferation. Among the common differential lncRNAs, *CYP3A29*, *AGMO*, *IGFALS*, *CES1*, *LEPR*, and *PLAC8* served as the target genes for MSTRG.35604.2, MSTRG.2011.1, MSTRG.26481.9, MSTRG.23976.1, MSTRG.40648.1, and MSTRG.27163.1.

### 3.4. Time-Series Analysis (STEM) of mRNAs and LncRNAs

**Figure 3:**
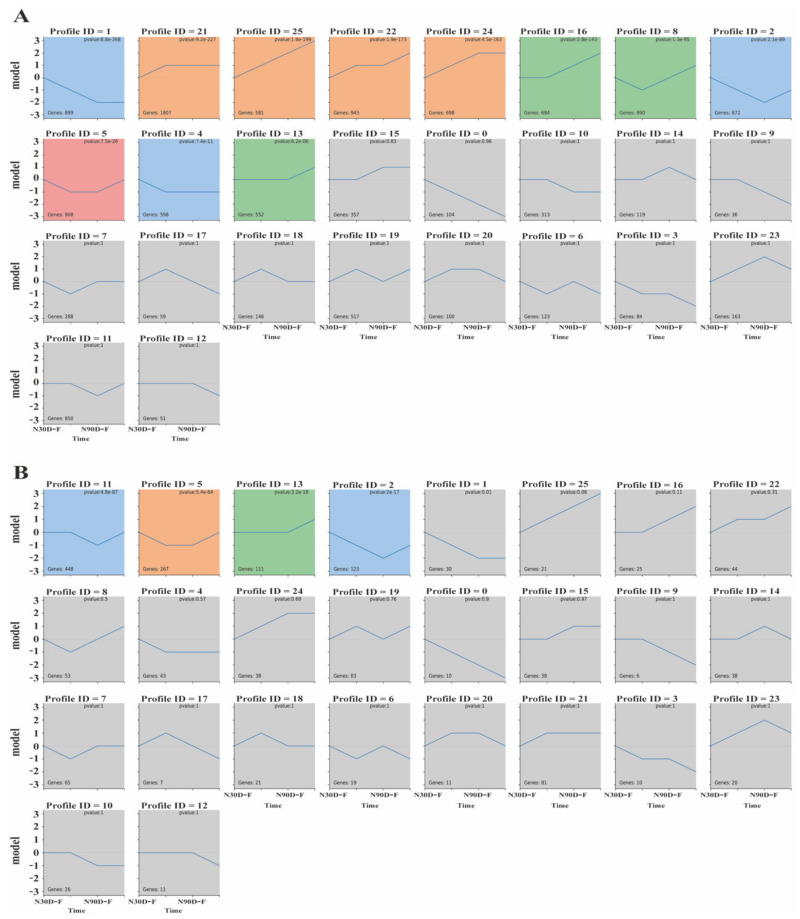
Short Time-series Expression Miner identified temporal expression profiles of (**A**) mRNAs and (**B**) lncRNAs. The top panel indicates the module number; numbers at the top right corner indicate *p*-value; numbers at the bottom left represents mRNAs or lncRNAs in each profile module.

**Figure 4:**
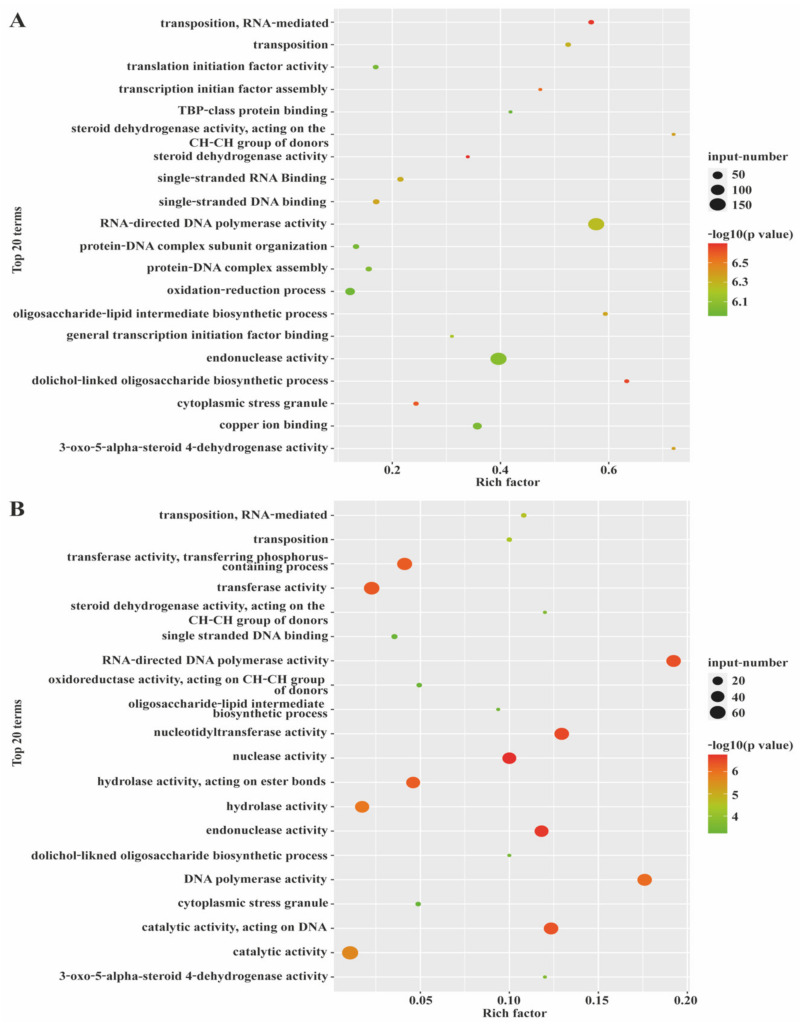
The distribution diagram of Gene Ontology (GO) functions of (**A**) mRNAs and (**B**) lncRNAs in STEM analysis.

The mRNA in subcutaneous adipose tissue was split into 11 modules. Module 25 showed a continuous expression pattern whereas modules 13, 14, 16, 21, 22, and 24 showed progressively increasing and decreasing transcription patterns (Figure 3A). The lncRNAs in subcutaneous adipose tissue were subdivided into four modules. Modules 2, 5, and 11 had expression patterns that were gradually reducing and then progressively increasing. Besides this, the expression pattern of lncRNAs in module 13 was progressively increasing (Figure 3B). By GO enrichment analysis, we found that the expression modules of mRNA (Figure 4A) and module 2 (Table S6) were mainly involved in carboxylic acid metabolism and mitotic cell cycle; module 5 (Table S7) participated in steroid dehydrogenase activity, oligosaccharide lipid intermediate biosynthesis, and transcriptional regulation. Meanwhile, for the lncRNA expression module (Figure 4B), the target genes of module 2 (Table S8) were mainly involved in lipid metabolism and metastatic activity; the target gene of module 5 (Table S9) mainly participated in transfusion enzymes, hydrolase and polymerase activity, catalytic activity, and lipid metabolism. After KEGG analysis, 91 (Table S10) and 307 (Table S11) KEGG pathway of lncRNAs and mRNAs were enriched in module 2, while 28 (Table S12) and 266 (Table S13) KEGG pathway of lncRNAs and mRNAs were enriched in module 5. These lncRNAs were participated in glycerophospholipid metabolism, AMPK signaling, steroid biosynthesis, and metabolic pathways. The mRNAs were taking part in the metabolic pathway, glycerolipid metabolism, cell cycle, and bile secretion. Interestingly, we found similar expression patterns of mRNAs and lncRNAs for modules 2 and 5. This finding may suggest that these modules have highly correlated in the development of fat in Ningxiang pigs. The mRNA (target genes of lncRNAs) and associated lncRNAs in modules 2 and 5 are listed in Table 4 and Table 5, respectively.

### 3.5. Co-Expression Network Analysis (WGCNA) of mRNA and LncRNAs

**Figure 5:**
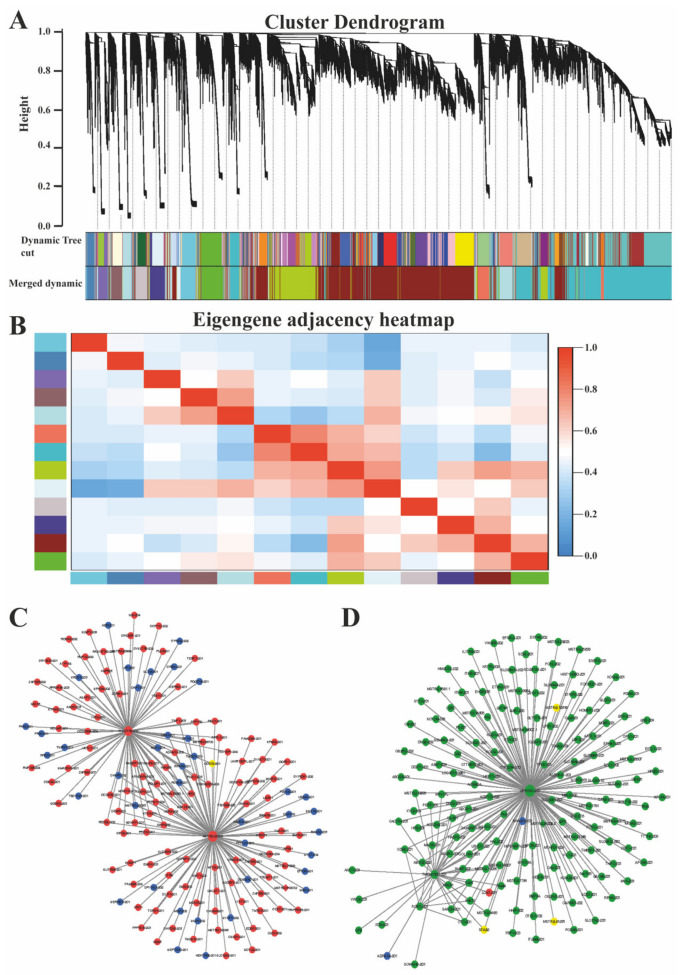
The weighted gene co-expression network analysis. (**A**) The hierarchical clustering dendrogram of lncRNA-mRNA co-expression modules in subcutaneous adipose tissue. Each branch represents a cluster of lncRNAs or mRNAs. Dynamic tree cut indicate original split module, and merged dynamic indicate final merged modules. (**B**) The hierarchical clustering dendrogram of module eigengenes and heatmap of adjacencies using WGCNA; red indicate positive correlation; blue indicate negative correlation. The co-expression network diagram of mRNAs and LncRNAs in the (**C**) darkred module and (**D**) darkturquoise module. Rhombic and circular nodes represent lncRNA and mRNA, respectively. Color indicates differential expression level: blue: no changes; red: up-regulation; green: down-regulation; yellow: up- and down-regulation.

A biological network of subcutaneous adipose tissue was built for four different physiological stages in Ningxiang pigs. On most nodes, mRNA and lncRNA were connected through several central nodes. The hub gene, the central node in the biological network, was explored by WGCNA analysis to discover the regulatory potential functional relationship between lncRNA and mRNA in subcutaneous adipose tissue and their mechanism (Figure 5A,B). By WGCNA analysis, 13 modules were obtained and 2 modules with the highest number of biological networks (Darkred and Darkturquoise module) were selected. Among them, *PHACTR2* was the hub gene of Darkred module (Figure 5C, Table S14); *GPRC5C* and *TMEM150C* were the hub gene of the Darkturquoise module (Figure 5D, Table S15). By GO enrichment analysis, we found that both darkred and darkturquoise were mainly involved in the biological process (68.42–72.06%), followed by molecular function (17.70–22.76%), and least by cellular components (5.00–13.68%). Furthermore, functional enrichment analysis revealed that co-expressed genes in the two largest modules were enriched in the hedgehog signaling pathway, Hippo signaling pathway, and primary bile acid biosynthesis.

### 3.6. RT-qPCR Quantification of mRNAs and lncRNAs

**Figure 6:**
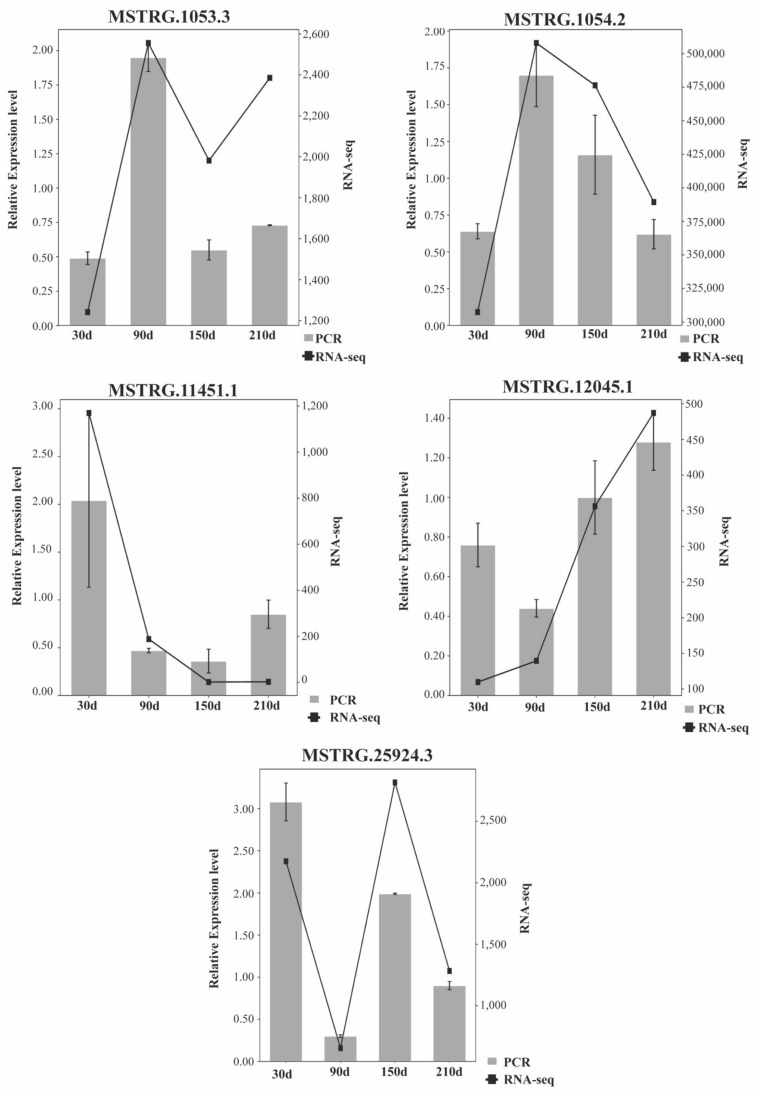
Expression patterns of MSTRG.1053.3, MSTRG.1054.2, MSTRG.11451.1, MSTRG.12045.1, and MSTRG.25924.3 compared to expression patterns in the RNA-seq.

**Figure 7:**
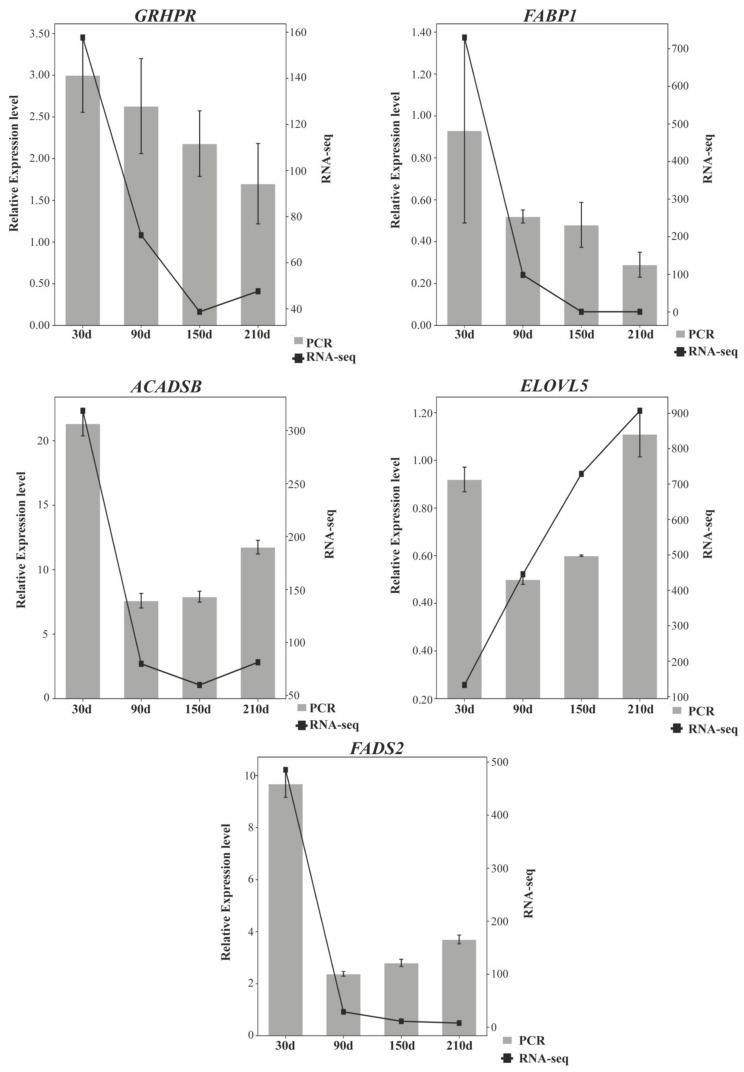
Transcription patterns of *GRHPR*, *FABP1*, *ACADSB*, *ELOVL5*, *FADS2* compared to expression patterns in the RNA-seq.

Five mRNAs—*GRHPR*, *FABP1*, *ACADSB*, *ELOVL5*, *FADS2*—and five lncRNAs—MSTRG.1053.3, MSTRG.1054.2, MSTRG.11451.1, MSTRG.12045.1, and MSTRG.25924.3—were randomly chosen for RNA-seq data reliable validation in Ningxiang pig. These genes were quantified via RT-qPCR at four developmental stages (piglet stage, nursery pig stage, early fattening stage, and late fattening stage). The lncRNAs RT-qPCR (Figure 6) and mRNAs RT-qPCR (Figure 7) results showed a concordance between the RT-qPCR data and RNA-seq, indicating that the RNA-seq data were reliable.

## 4. Discussion

### 4.1. Differentially Expressed lncRNAs and mRNAs

In general, because of their ability to engage with protein, RNA, and even DNA, lncRNAs can mediate gene regulation through a variety of mechanisms. LncRNAs can act as signals to stimulate or repress transcriptional processes, as an epigenetic moderator, or even as scaffold that interact with other protein to produce ribonucleoprotein complex [26,27,28]. The lncRNAs in subcutaneous adipose tissue of Ningxiang pigs may affect adipose tissue development or lipid metabolism. In this study, a total of 10,084 DEmRNAs and 931 DElncRNAs were identified. mRNAs were most differentially expressed in the piglet stage (30 days) compared to other development stages (90, 150, and 210 days). This situation suggests that the expression of mRNAs in the piglet stage may be significant for the development of subcutaneous adipose tissue. We suggest that changes and differences in dietary regimen during the sucking and weaning periods in Ningxiang pig may influence digestion, metabolism, and the development of subcutaneous adipose tissue, thus influencing the different expression numbers of mRNAs [29]. The lncRNAs presented the highest upregulation in the late fattening stage (210 days), which may indicate that lncRNAs have higher regulatory activity at this stage than at other stages. In the late fattening stage, the weight of Ningxiang pigs tended to increase as the pigs were approaching sexual maturity. The findings of the study showed that lncRNAs were mainly enriched in transferase activity, DNA polymerase activity, nucleotide transaminase activity, nuclease activity, endonuclease activity, and hydrolase activity operating in ester bonds. The activities mentioned above could be the reason why DElncRNAs are highly up-regulated in the late fattening stage.

From the Venn diagrams, we founded that *FADS2*, *CYP3A29*, *CYP2C42*, *PLCB4*, *LEPR*, *CES1D* are involved in lipid metabolism, while *IGF2*, *IGFALS*, and *FGFR4* are involved in cell differentiation and proliferation in DEmRNAs. Among the DElncRNAs, we found that *CYP3A29*, *IGFALS*, *CES1*, *LEPR*, and *PLAC8* served as target genes for MSTRG.35604.2, MSTRG.26481.9, MSTRG.23976.1, MSTRG.40648.1, and MSTRG.27163.1. The insulin-like growth factor binding protein, acid-labile subunit (*IGFALS*), participated in the circulation of insulin-like growth factor and is significant in growth and development [30]. The leptin receptor (*LEPR*) is an active participant in body weight regulation, fat mobilization, and energy homeostasis in animals [31]. Carboxylesterase 1 (*CES1*), a member of the serine esterase family, primarily participated in adipose tissue lipolytic activity, and is linked to obesity or weight loss [32]. The placenta-specific 8 (*PLAC8*) is an upstream regulator of brown adipose tissues [33]. Brown fat is a primary effector of additive thermogenesis, in which brown adipocytes oxidize fatty acids to produce heat in response to cold conditions [34]. The current findings propose that DElncRNAs and DEmRNAs may be associated with lipid metabolism. This phenomenon may affect the growth and meat quality of fatty pig bread such as Ningxiang pigs. Functional studies should be conducted on the DElncRNAs and DEmRNAs identified in this study to obtain insight into the role and regulation of these DElncRNAs and DEmRNAs. An in-depth understanding of the functional role of these genes could improve the productivity of Ningxiang pigs. 

### 4.2. Time-Series Analysis (STEM) of mRNAs and lncRNAs

We are aware that most biological processes are dynamic, and therefore sequential (time-series) experiments are significant for researchers to understand these processes [35]. The Short Time-series Expression Miner (STEM) enables scholars to analyze short time series expression data and incorporated it with gene ontology to explain the data biologically [22]. In the present study, STEM analysis revealed dynamic and similar expression patterns between mRNAs and lncRNAs during the development of subcutaneous adipose tissue in Ningxiang pigs. We found that both modules 2 and 5 showed similar expression mode in both mRNA and lncRNA. KEGG pathway analysis revealed that genes in both mRNA and lncRNA were mainly participated in steroid biosynthesis, glycosphingolipid biosynthesis, metabolic pathways, and glycerolipid metabolism. In modules 2 and 5, the expression levels of mRNAs and lncRNAs were higher at 150 and 210 days after birth. This may be due to the active fatty acid, sterol, steroid, and lipid-related metabolism activity in the subcutaneous adipose tissue during the early fattening stage (150 days) and late fattening stage (210 days). 

Interferon lambda receptor 1 (*IFNLR1*) is a cytokine that regulates infection in the liver, brain, and gastrointestinal tract in mice [36]. *IFNLR1* has been declared to be a better therapeutic approach for porcine epidemic diarrhea in piglets [37]. Adenylyl cyclase type 9 (*ADCY9*) catalyzes the formation of cAMP in response to G protein-coupled receptor activation, which contributes to the corticosteroids and beta-adrenergic receptor signaling cascade. Previous study has demonstrated that inactivation of *ADCY9* in the absence of cholesteryl ester transfer protein may increase the adipose tissue volume, feed efficiency, and weight gain [38]. Sphingomyelin synthase 1 (*SGMS1*) is a protein-coding gene that is involved in sphingolipid metabolism. Gene Ontology annotations show that *SGMS1* is associated ceramide cholinephosphotransferase activity. *SGSM1* regulates the sphingomyelin content in the plasma membrane and is participated in lipid raft formation [39]. 2,4-Dienoyl-CoA Reductase 1 (*DECR1*) is a nuclear-encoded mitochondrial enzyme that is primarily engaged in the metabolism of unsaturated fatty acid enoyl-CoA ester [40]. Besides this, *DECR1* is associated with the β-oxidation of polyunsaturated fatty acids, and the regulation of lipid deposition [41]. Depletion of *DECR1* may lead to high levels of serum acylcarnitine, as a result of incomplete oxidation of unsaturated fatty acids. From the results obtained, it is clear that the target genes (mRNAs) of lncRNAs are mainly involved in lipid metabolism in the subcutaneous adipose tissue. Subcutaneous adipose tissues may play a vital regulatory role in lipid metabolism during the early fattening stage and late fattening stage. Furthermore, lipid metabolism ensures normal homeostasis in piglets, especially during the fattening stage. Therefore, optimal lipid metabolism may strengthen the growth performance and survival of Ningxiang piglets.

### 4.3. Co-Expression Network Analysis of mRNAs and LncRNAs

By the mRNA–lncRNA co-expression network analysis, *PHACTR2* was determined as the hub gene of the darkred module, while *GPRC5C* and *TMEM150C* were identified as the hub genes of the darkturquoise module. The phosphatase and actin regulator 2 (*PHACTR2*) is a protein-coding gene that is related to actin binding and protein phosphatase inhibitor activity. Previous studies have indicated that the *PHACTR* family is highly transcribed in the nervous system and suggested that this protein-coding gene may regulate the binding of protein phosphatase 1 to cytoplasmic actin. However, functional characterization of *PHACTR2* remains scarce. Interestingly, we found that *PHACTR2* is a hub gene in co-expression network analysis [42]. This result caught our attention, and we intend to further investigate the functional and regulatory role of *PHACTR2* in subcutaneous adipose tissue. A g-protein coupled receptor family C group 5 (*GPRC5C*) is a protein-encoding gene that mediates the cellular effects of retinoic acid on G protein signal transduction concatenation. In addition, *GPRC5C* plays a vital role in axon guidance, transmitter release, neuronal development, cell proliferation, and glucose-stimulated insulin secretion [43]. The knockdown of *GPRC5C* significantly influences the regulatory functions of the hematopoietic system and metabolic homeostasis pathways [44,45]. As *GPRC5C* became a hub gene in co-expression studies, we speculate that *GPRC5C* may be essential and play a regulatory role in maintaining normal metabolic homeostasis in the subcutaneous adipose tissue of Ningxiang pigs. Transmembrane protein 150C (*TMEM150C*), also known as tentonin 3, is a transmembrane protein component of a mechanosensitive ion channel that is crucial for blood pressure regulation, touch, pain, and hearing [46]. Previous studies have found that *TMEM150C* may affect phospholipid homeostasis at the plasma membrane. In addition, *TMEM150C* may amend the chemical composition of the plasma membrane and change its tension and rigidity. Lipids can influence the function of ion channel directly or have subtle effects by reassigning channels to membrane subdomains [47]. These pivotal genes may play a vital role in subcutaneous adipose tissue by regulating downstream genes to maintain ideal metabolism activity and homeostasis, which in turn promote the development of Ningxiang piglets. 

In addition to hub genes, the hedgehog signaling pathway, Hippo signaling pathway, and primary bile acid biosynthesis is also enriched and take part in the development of subcutaneous adipose tissue. Hedgehog signaling pathway is a highly conserved pathway involved in the regulation of invertebrate and vertebrate organogenesis and tissue homeostasis [48]. Hedgehog signaling has been declared to influence the adipogenic differentiation of preadipocytes [49]. Previous studies revealed that the inactivation of hedgehog signaling may promote lipid accumulation and that the lipase brummer (Bmm) works downstream as a smoothened (Smo) in hedgehog signaling to accelerate the lipolysis in adipose tissue [50]. In the present study, abundance of genes was enriched in the hedgehog signaling pathway; therefore, we speculate that the hedgehog signaling pathway is crucial in maintaining the lipid accumulation in the subcutaneous adipose tissue of Ningxiang piglet. The Hippo signaling pathway has arisen as a significant regulator of normal organogenesis in vertebrates [51]. Furthermore, the Hippo signaling pathway is also participated in the regulation of adipogenesis, adipose cell differentiation and proliferation [52]. Previous studies have reported that the Lats2 in the Hippo signaling pathway may influence the equilibrium of differentiation and proliferation during adipogenesis [53]. In addition, Hippo kinase (MST1/2) is a positive enhancer for adipogenesis. The transcription levels of MST and SAV1 in the Hippo signaling pathway are significantly increased during the differentiation of preadipocytes into adipocytes [52]. Bile acids are significant elements of lipid metabolism and are the end product of cholesterol catabolism. In addition to lipid metabolism, bile acids also involved in the regulation of energy metabolism. Cholesterol homeostasis is retained through catabolism, which primarily involves the conversion of cholesterol to hydrophilic bile acids [54]. Previous studies have reported that the bile acids may be successful in combating obesity and metabolic disease, and that higher brown adipose tissue activity may increase energy [55]. The role of bile acids in energy metabolism may help to improve the condition of piglets when exposed to cold weather. From the results, it appears that the hedgehog signaling pathway, Hippo signaling pathway and primary bile acid biosynthesis may play a vital role in maintaining normal homeostasis in Ningxiang piglets. Adipose tissue is an important organ participated in lipid metabolism and fat deposition in both invertebrate and vertebrate. The piglets are quite circumscribed and fragile and require a normal homeostasis to maintain proper growth. Therefore, an in-depth understanding of the co-expression between mRNAs and lncRNAs in subcutaneous adipose tissue could provide some clues and basic knowledge on how to improve homeostasis, and thus enhance the growth and survival of Ningxiang piglets.

## 5. Conclusions

In summary, this study reports the functional network and expression profiles of mRNAs and lncRNAs in the subcutaneous adipose tissue of Nixngxiang piglets at four developmental phases (piglet stage, 30 days after birth; nursery pig stage, 90 days after birth; early fattening stage, 150 days after birth; and late fattening stage, 210 days after birth). A total of 2872 novel lncRNAs were identified, classified as intergenic (1263), antisense (766), bidirectional (29), sense exon overlapping (769), and sense intron overlapping lncRNAs (45). We identified a total of 10084 DEmRNAs and 931 DElncRNAs and found that most DEmRNAs were up-regulated at the piglet stage and down-regulated at the late fattening stage. STEM and WGCNA analysis revealed complex interactions between mRNAs and lncRNAs, suggesting that lncRNAs are significant regulatory components of mRNAs. We found that modules 2 and 5 exhibit similar expression modes on both mRNA and lncRNA. They are mainly involved in steroid biosynthesis, glycosphingolipid biosynthesis, metabolic pathways, and glycerolipid metabolism. In both modules 2 and 5, the transcription levels of mRNAs and lncRNAs were highest in both early and late fattening stage. This may be explained by the active metabolic activities related to fatty acid, sterol, steroid, and lipid in the subcutaneous adipose tissue during the early and late fattening stage. The results of this study lay the foundations for the study of lncRNAs in Ningxiang pigs and provide new insights into the functions of lncRNAs in subcutaneous adipose tissue at different developmental stages. Functional studies of the detected DEmRNAs and DElncRNAs should be carried out to obtain deeper information about the regulatory properties of these genes. The knowledge gained may help to improve the growth performance and ensure the survival of Ningxiang pigs.

## Figures and Tables

**Table 1:** Forward and reverse primers used for gene quantification by RT-qPCR.

Name	Sequence (5’ to 3’)
MSTRG.1053.3-F	ACTTGGGAAGAAAGCAATTTTAAGA
MSTRG.1053.3-R	TGTAGTCCCAGCTACTCGGG
MSTRG.11451.1-F	AGACATCCGAGCCTGGGATA
MSTRG.11451.1-R	CGTTTCAGAAAGCGTTGGAAGT
MSTRG.25924.3-F	AGACATCCGAGCCTGGGATA
MSTRG.25924.3-R	GTGTCGAGGGCTGACTTTCA
MSTRG.12045.1-F	CGCTGAGCTGTTGGGTATGA
MSTRG.12045.1-R	AGCGTTGGGAAGTGCTCTTT
MSTRG.1054.2-F	TGTAGTCCCAGCTACTCGGG
MSTRG.1054.2-R	ACAGGGTCTCGCTATGTTGC
*GRHPR*-F	GCAGTGGGATTCCGATGAG
*GRHPR*-R	CGTGGTGTCGCTTCTTGATTTC
*FADS2*-F	CAGCACGATTACGGCCATCT
*FADS2*-R	AGATGTTGGGCTTGGCATGA
*FABP1*-F	GTCTGCCCCGACGAACTCAT
*FABP1*-R	CTCATTCTGGACGACCTTGGA
*ELOVL5*-F	CATCCTGCGCAAGAACAACC
*ELOVL5*-R	GGGATGGATGACAGACCGTAG
*ACADSB*-F	GGAAGAGTCCAACGCATTCA
*ACADSB*-R	GAGGAGGTTGCTCTCTGACG

**Table 2:** Go enrichment analysis of mRNAs in closed groups (30d vs. 90d, 30d vs. 150d, and 30d vs. 210d).

Groups	mRNAs	lncRNAs
30d vs. 90d (upregulated)	-Tissue and organ morphology development -Epithelial and endothelial cell proliferation -Extracellular matrix -Cell adhesion -Calcium ion binding	-Zinc ion binding -Transition metal element ion binding -Transcription factor complex -Steroid hormone receptor activity
30d vs. 150d (upregulated)	-Cell link assembly regulation -Smooth muscle cell proliferation regulation -Negative feedback regulation of angiogenesis -Collagen fiber tissue development -Protein folding	-Transcription factor complex -Ligand activated transcription factor activity -Steroid hormone receptor activity -Thyroid hormone-mediated signaling pathway -Nuclear receptor activity
30d vs. 210d (upregulated)	-Endothelial cell proliferation -Negative regulation of vascular development -Regulation of cell junction assembly -Collagen fiber tissue -Adsorption of cells and substrates	-Lipid biosynthesis regulation -Fatty acid oxidation regulation -Fatty acid metabolism regulation -Acyl-CoA biosynthesis -Male gonadal development -Ion homeostasis
30d vs. 90d (downregulated)	-Steroid dehydrogenase activity -Steroid metabolism -RNA-guided DNA polymerase activity -Organic acid biosynthesis -Cellular amino acid metabolism -Catalytic activity on DNA	-Catalytic activity -Hydrolase activity -Myosin filaments -Myosin complex
30d vs. 150d (downregulated)	-Steroid hydroxylase and dehydrogenase activity -Cholesterol reversal transport -Lipoprotein complex -Lipoprotein particles	-DNA packaging complex -Protein-DNA complex -Nucleosome -Protein heterodimerization activity -21-carbon steroid hormone metabolism -Androgen dehydrogenase activity -Ketosteroid monooxygenase activity
30d vs. 210d (downregulated)	-Triglyceride homeostasis -Sterol biosynthesis and transport -High-density lipoprotein particles -Dihydroxy acid metabolism process	-Protein-DNA complex -Protein heterodimerization activity -Nucleosome

**Table 3:** Go enrichment analysis of mRNAs in consecutive groups (30d vs. 90d, 90d vs. 150d, and 150d vs. 210d).

Groups	mRNAs	lncRNAs
30d vs. 90d (upregulated)	-Tissue and organ morphology development -Epithelial and endothelial cell proliferation -Extracellular matrix -Cell adhesion -Calcium ion binding	-Zinc ion binding -Transition metal element ion binding -Transcription factor complex -Steroid hormone receptor activity
90d vs. 150d (upregulated)	-Protein folding -Chaperone-mediated protein folding	-RNA binding -Positive regulation of epithelial cell -proliferation -Male gonadal development -Cell response to peptides
150d vs. 210d (upregulated)	-Sterol homeostasis and transport, -Lipoprotein particles, -High-density lipoprotein particles, -Cholesterol homeostasis and transport activity	-Transferase activity -DNA polymerase activity -Nucleotide transaminase activity -Nuclease activity -Endonuclease activity -Hydrolase activity acting on ester bonds
30d vs. 90d (downregulated)	-Steroid dehydrogenase activity -Steroid metabolism -RNA-guided DNA polymerase activity -Organic acid biosynthesis -Cellular amino acid metabolism -Catalytic activity on DNA	-Catalytic activity -Hydrolase activity -Myosin filaments -Myosin complex
90d vs. 150d (downregulated)	-Transferase activity -DNA polymerase activity -Nuclease activity -Nucleotide transferase activity -Hydrolase activity acting on ester bonds -Steroid dehydrogenase activity	-Protein-DNA complexes -Protein heterodimers and dimer activity -Nucleosomes -DNA packaging complexes and chromatin parts
150d vs. 210d (downregulated)	-Protein folding -Chaperone-mediated protein folding -Protein stability regulation	-Thyroid development -Signal pathway mediated by thyroid hormone -Negative regulation of transcription initiation by RNA polymerase II promoter

**Table 4:** The mRNA (target genes of lncRNAs) and associated lncRNAs in Module 2.

mRNA	mRNA Functional Description	Associated lncRNAs
*IFNLR1*	Encoding receptors for cytokine ligands IFNL2 and IFNL3 and mediating their antiviral activity	MSTRG.23976.1 MSTRG.12196.6 MSTRG.26481.9 MSTRG.42001.1
*TARBP1*	S-adenosyl-L-methionine-dependent methyltransferase can methylate RNA molecules, such as tRNA	MSTRG.9023.5 MSTRG.11929.3
*ADCY9*	Adenylate cyclase can catalyze the signal molecule cAMP to respond to the activation of G protein-coupled receptors.	MSTRG.18828.1 MSTRG.15237.1

**Table 5:** The mRNA (target genes of lncRNAs) and associated lncRNAs in Module 5.

mRNA	mRNA Functional Description	Associated lncRNAs
*SGMS1*	Encoding ceramide choline phosphotransferase, which catalyzes the reversible transfer of part of phosphocholine during the biosynthesis of sphingomyelin. In the forward reaction, the head-end group of phosphatidylcholine can be transferred to ceramide to form ceramide phosphorylcholine and diacylglycerol. In the reverse reaction, the product obtained is the opposite of the uninhibited reaction. The direction of the reaction is related to the levels of ceramide and diacylglycerol in the Golgi membrane	MSTRG.1058.1
*DECR1*	Encoding 2,4-dienoyl-CoA reductase 1, involved in β-oxidation and metabolism of unsaturated fatty enoyl-CoA esters.	MSTRG.24004.1
*HS3ST4*	Encoding heparan sulfate-glucosamine 3-sulfotransferase 4	MSTRG.25084.1 MSTRG.8247.1 MSTRG.8043.1 MSTRG.7789.1 MSTRG.7789.1 MSTRG.7605.3 MSTRG.6591.1 MSTRG.45142.4
*ZNF709*	Encoding zinc finger protein 709	MSTRG.38367.1 MSTRG.24004.1 MSTRG.25790.1 MSTRG.27767.1 MSTRG.23025.1 MSTRG.1895.1 MSTRG.24004.1

## Data Availability

The data presented in this study are available in the supplementary material.

## Supplementary Materials

The following are available online at https://www.mdpi.com/article/10.3390/biology10080726/s1. Table S1: The statistics of raw and clean read after quality assessment. Table S2: Statistic of mapping to the Ningxiang pig reference genome. Table S3: Identified novel lncRNAs in subcutaneous adipose tissues. Table S4: Total differentially expressed mRNAs of subcutaneous adipose tissue. Table S5: Total differentially expressed lncRNAs of subcutaneous adipose tissue. Table S6: GO enrichment in mRNAs module profile 2 of STEM. Table S7: GO enrichment in mRNAs module profile 5 of STEM. Table S8: GO enrichment in lncRNAs in module profile 2 of STEM. Table S9: GO enrichment in lncRNAs in module profile 5 of STEM. Table S10: KEGG enrichment in lncRNAs module profile 2 of STEM. Table S11: KEGG enrichment in mRNAs in module profile 2 of STEM. Table S12: KEGG enrichment in lncRNAs module profile 5 of STEM. Table S13: KEGG enrichment in mRNAs in module profile 5 of STEM. Table S14: GO enrichment in darkred module of WGCNA. Table S15: GO enrichment in darkturquoise module of WGCNA.
