# Supplementary material for: Integrated Analysis of lncRNA and mRNA in Subcutaneous Adipose Tissue of Ningxiang Pig
Source: Biology (Basel). 2021 Jul 29;10(8):726. doi: 10.3390/biology10080726 (PMC8389317; doi:10.3390/biology10080726)
Supplement: Supplementary file 1 [file biology-10-00726-s001.zip › Table S1.pdf]

Table S1. The statistics of raw and clean read after quality assessment.

Sample	Raw reads	Raw bases	Clean reads	Clean bases	Error rate (%)	Q20 (%)	Q30 (%)	GC content (%)
30-1	121,565,982	18,356,463,282	119,964,536	16,633,665,718	0.0238	98.44	95.45	49.39
30-2	99,434,348	15,014,586,548	98,170,604	13,702,767,066	0.0248	98.05	94.36	51.15
30-3	122,671,378	18,523,378,078	121,069,786	16,547,185,848	0.0239	98.37	95.35	50.37
90-1	115,376,876	17,421,908,276	112,769,622	14,682,375,514	0.0239	98.32	95.41	53.59
90-2	105,102,906	15,870,538,806	103,159,740	11,583,593,548	0.0238	98.39	95.52	53.96
90-3	101,557,822	15,335,231,122	99,679,884	13,078,904,949	0.0237	98.43	95.60	52.49
150-1	88,875,218	13,420,157,918	87,647,816	12,089,975,463	0.0239	98.40	95.41	52.85
150-2	95,340,350	14,396,392,850	93,706,234	12,663,602,608	0.0238	98.38	95.46	54.11
150-3	86,505,826	13,062,379,726	85,444,152	11,702,143,233	0.0236	98.50	95.65	52.68
210-1	125,482,560	18,947,866,560	123,227,520	16,033,805,832	0.0240	98.32	95.26	51.51
210-2	114,741,162	17,325,915,462	113,053,758	15,305,531,378	0.0239	98.42	95.39	48.77
210-3	109,012,944	16,460,954,544	107,459,030	14,479,629,717	0.0240	98.32	95.25	49.88
