# Supplementary material for: Integrated Analysis of lncRNA and mRNA in Subcutaneous Adipose Tissue of Ningxiang Pig
Source: Biology (Basel). 2021 Jul 29;10(8):726. doi: 10.3390/biology10080726 (PMC8389317; doi:10.3390/biology10080726)
Supplement: Supplementary file 1 [file biology-10-00726-s001.zip › Table S2.pdf]

**Table S2.** Statistic of mapping to the Ningxiang pig reference genome (accession number: PPJNA531381).

| Sample | Total reads | Total mapped | Multiple mapped | Unique mapped |
|---|---|---|---|---|
| 30-1 | 119,964,536 | 112,944,829（94.15%） | 5,055,828（4.21%） | 107,889,001（89.93%） |
| 30-2 | 98,170,604 | 92,110,906（93.83%） | 4,508,581（4.59%） | 87,602,325（89.23%） |
| 30-3 | 121,069,786 | 113,802,102（94.00%） | 4,826,332（3.99%） | 108,975,770（90.01%） |
| 90-1 | 112,769,622 | 104,175,961（92.38%） | 7,447,982（6.60%） | 96,727,979（85.77%） |
| 90-2 | 103,159,740 | 95,119,968（92.21%） | 6,073,279（5.89%） | 89,046,689（86.32%） |
| 90-3 | 99,679,884 | 93,157,059（93.46%） | 6,025,997（6.05%） | 87,131,062（87.41%） |
| 150-1 | 87,647,816 | 80,900,635（92.30%） | 5,282,770（6.03%） | 75,617,865（86.27%） |
| 150-2 | 93,706,234 | 86,265,325（92.06%） | 7,254,082（7.74%） | 79,011,243（84.32%） |
| 150-3 | 85,444,152 | 78,572,372（91.96%） | 4,550,797（5.33%） | 74,021,575（86.63%） |
| 210-1 | 123,227,520 | 103,379,434（94.26%） | 6,055,130（5.52%） | 97,324,304（88.74%） |
| 210-2 | 113,053,758 | 107,849,447（94.74%） | 6,120,683（5.38%） | 101,738,764（89.36%） |
| 210-3 | 107,459,030 | 102,328,874（94.74%） | 6,357,426（5.89%） | 95,971,448（88.85%） |
