# Supplementary figures and images for: Integrated Analysis of lncRNA and mRNA in Subcutaneous Adipose Tissue of Ningxiang Pig
Source: Biology (Basel). 2021 Jul 29;10(8):726. doi: 10.3390/biology10080726 (PMC8389317; doi:10.3390/biology10080726)

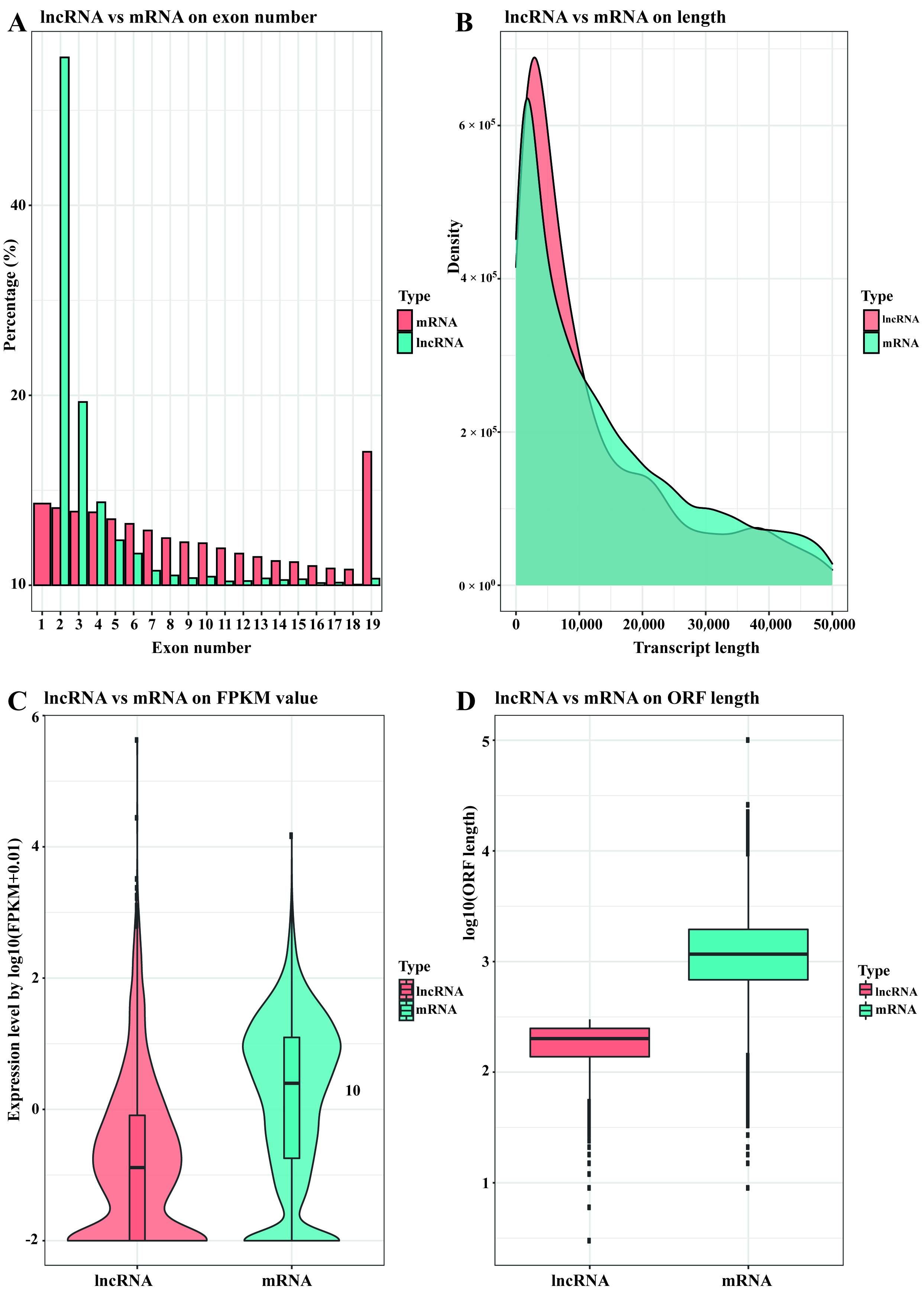

Supplement: Supplementary file 1 [file biology-10-00726-s001.zip › Figure 1.tif]

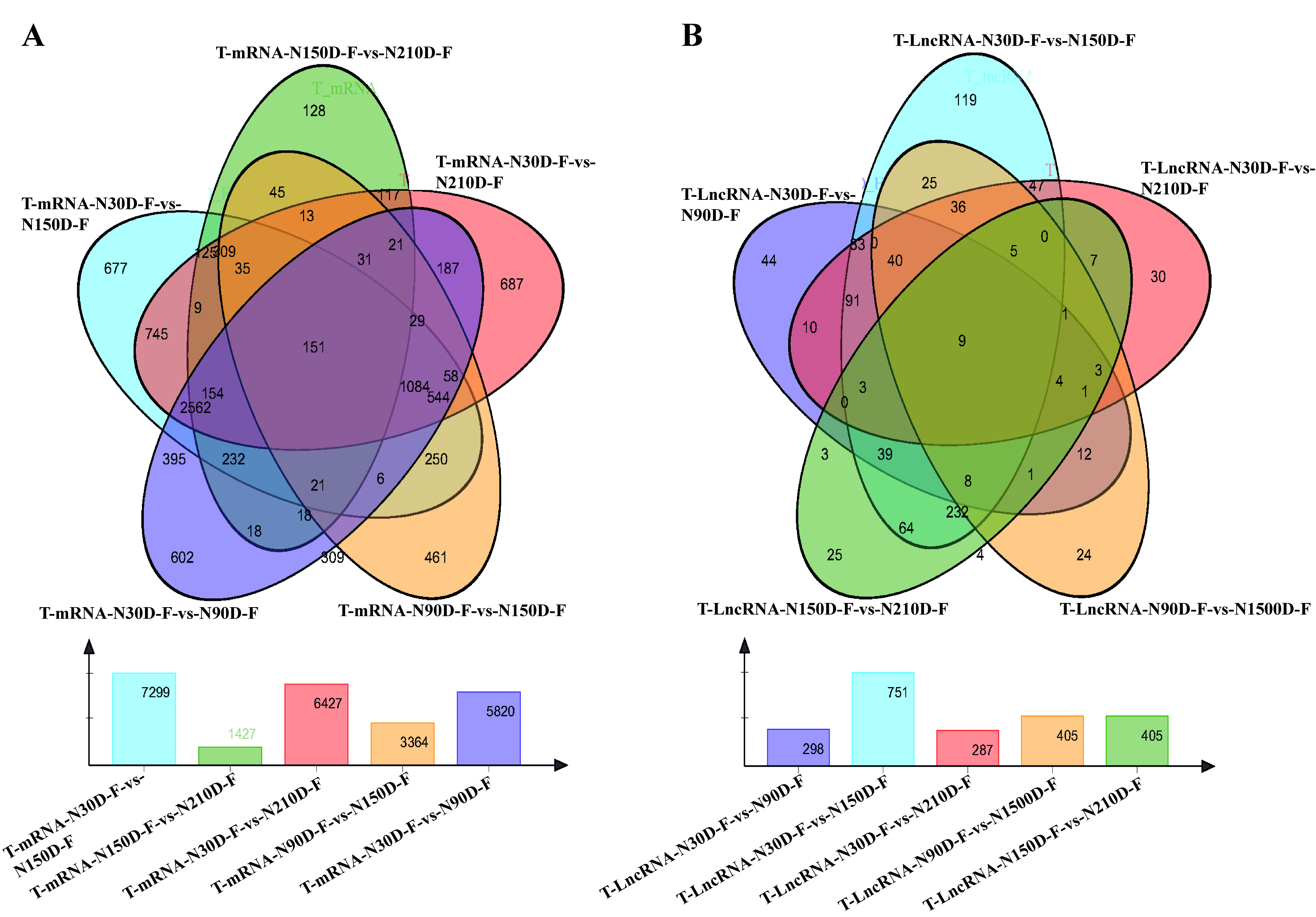

Supplement: Supplementary file 1 [file biology-10-00726-s001.zip › Figure 2.tif]

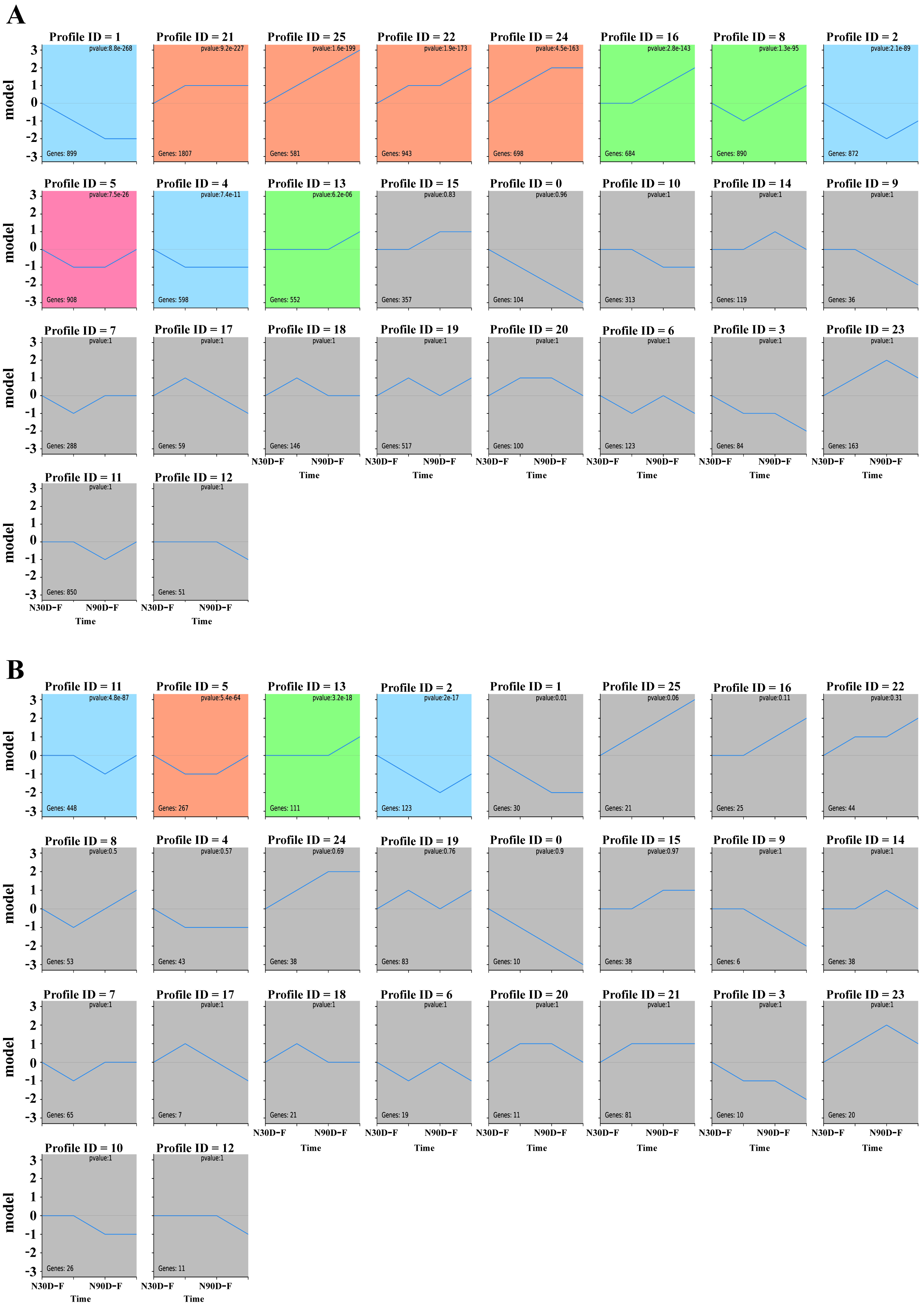

Supplement: Supplementary file 1 [file biology-10-00726-s001.zip › Figure 3.tif]

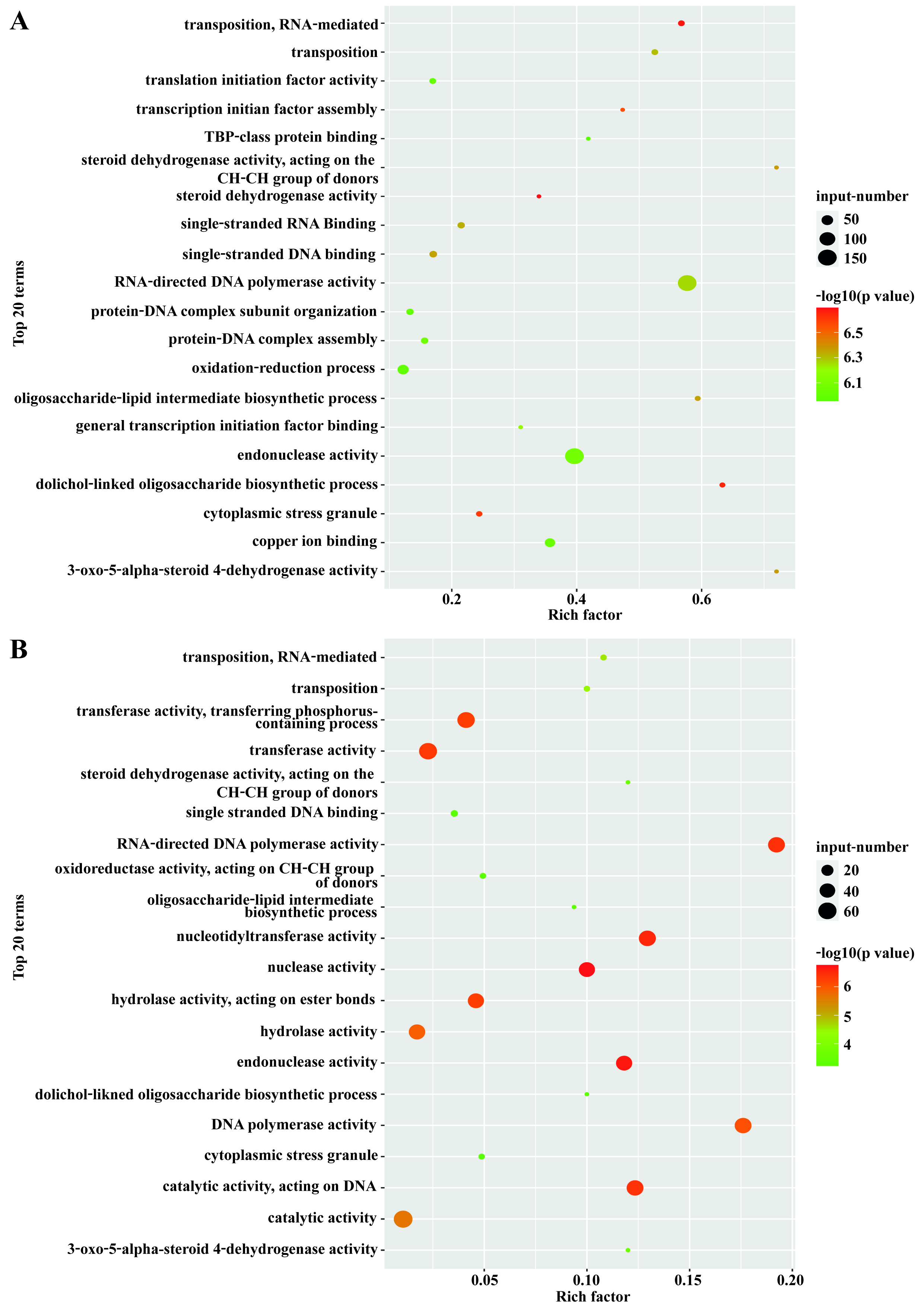

Supplement: Supplementary file 1 [file biology-10-00726-s001.zip › Figure 4.tif]

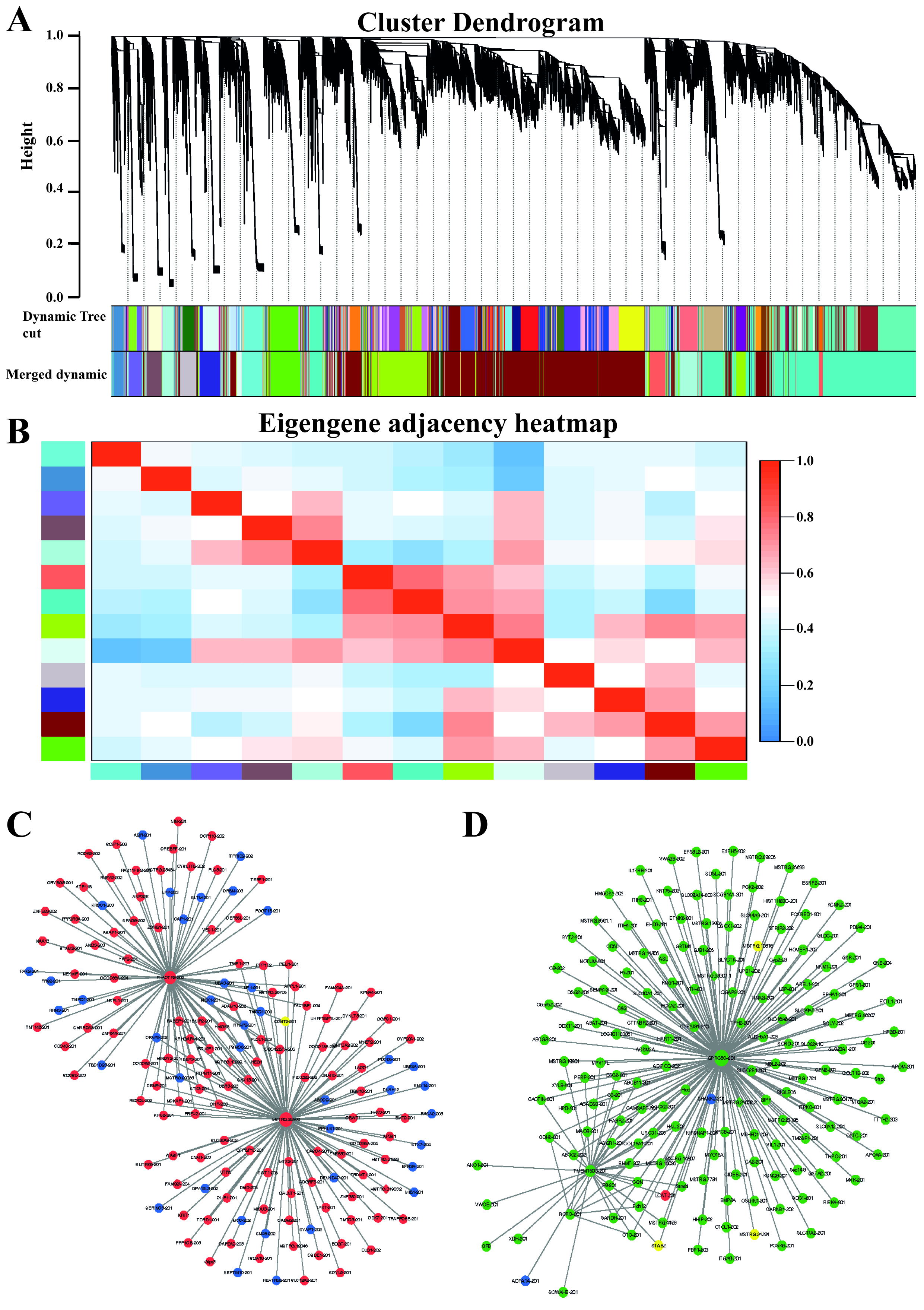

Supplement: Supplementary file 1 [file biology-10-00726-s001.zip › Figure 5.tif]

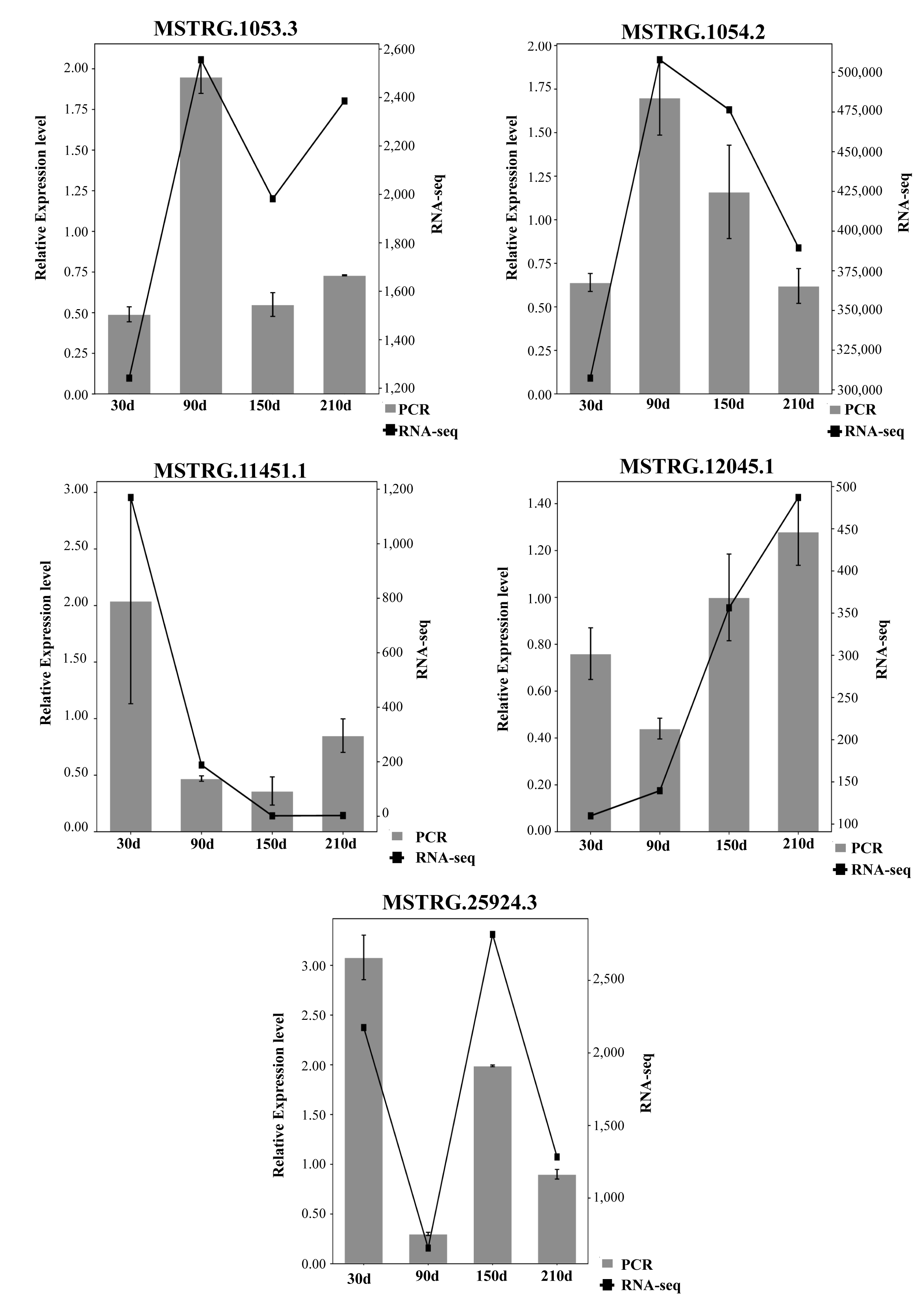

Supplement: Supplementary file 1 [file biology-10-00726-s001.zip › Figure 6.tif]

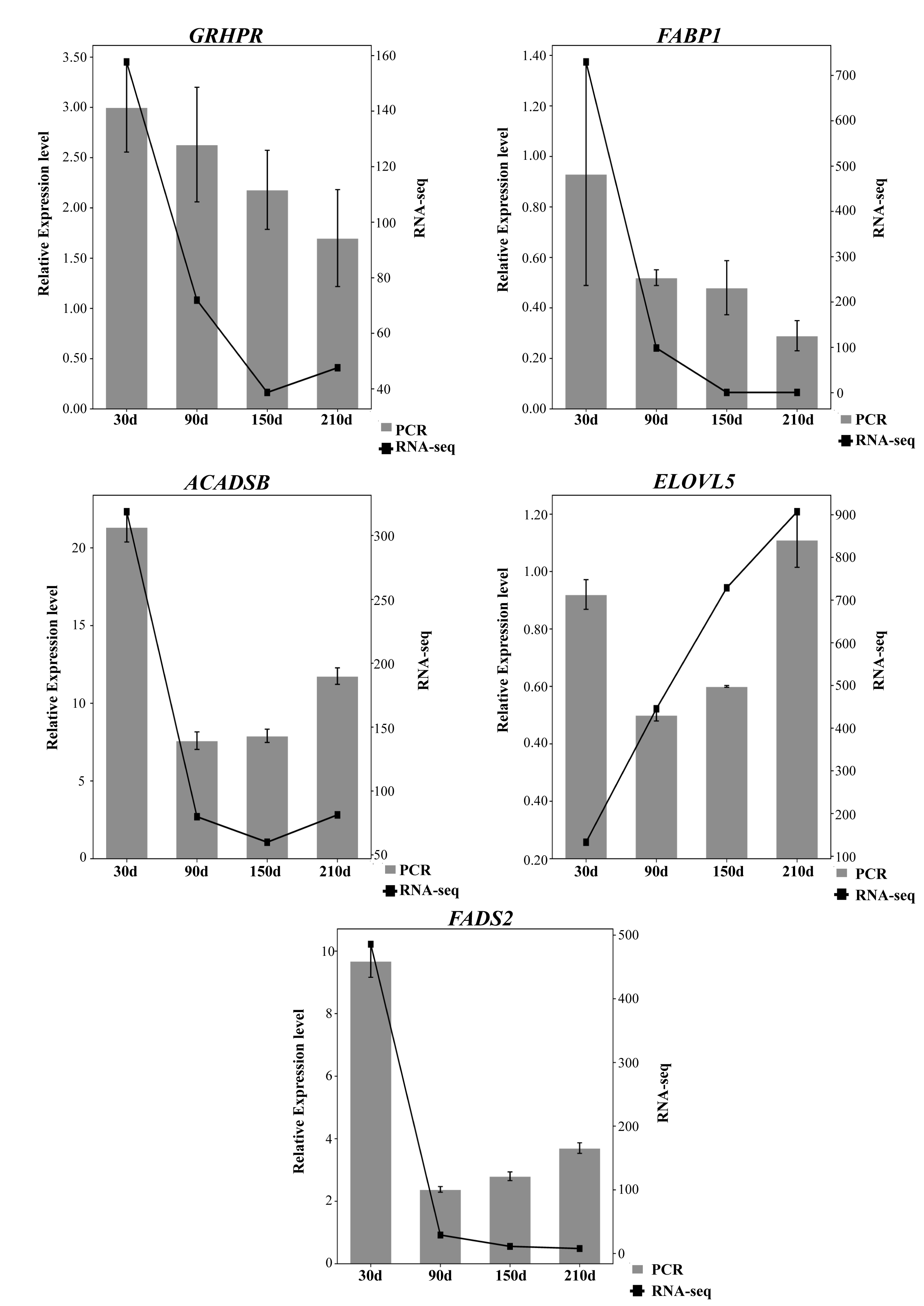

Supplement: Supplementary file 1 [file biology-10-00726-s001.zip › Figure 7.tif]
